# The relationship between HbA1c & atrial fibrillation after off-pump coronary artery bypass surgery in diabetic patients

**DOI:** 10.12669/pjms.321.8588

**Published:** 2016

**Authors:** Suleyman Surer, Mustafa Seren, Onur Saydam, Ali Bulut, Ugursay Kiziltepe

**Affiliations:** 1 Suleyman Surer, Chief Resident, Diskapi Yildirim Beyazit Training & Research Hospital, Department of Cardiovascular Surgery, Diskapi-Altindag/Ankara, Turkey; 2 Mustafa Seren, Chief Resident, Diskapi Yildirim Beyazit Training & Research Hospital, Department of Cardiovascular Surgery, Diskapi-Altindag/Ankara, Turkey; 3 Onur Saydam, Chief Resident, Diskapi Yildirim Beyazit Training & Research Hospital, Department of Cardiovascular Surgery, Diskapi-Altindag/Ankara, Turkey; 4 Ali Bulut, Chief Resident, Diskapi Yildirim Beyazit Training & Research Hospital, Department of Cardiovascular Surgery, Diskapi-Altindag/Ankara, Turkey; 5 Ugursay Kiziltepe, Professor of Cardiovascular Surgery, Diskapi Yildirim Beyazit Training & Research Hospital, Department of Cardiovascular Surgery, Diskapi-Altindag/Ankara, Turkey

**Keywords:** HbA1c, Off-pump coronary surgery, Atrial fibrillation

## Abstract

**Objective:**

Diabetes mellitus is recognized as a risk factor for mortality and morbidity after coronary bypass grafting. We aimed to determine the association between preoperative hemoglobin HbA1c and AF after isolated off-pump coronary bypass grafting (OPCAB).

**Methods:**

The seventy-two diabetic patients undergoing isolated off-pump coronary bypass grafting were retrospectively analyzed for AF. They were divided into; Low (4.8–5.4%), Medium (5.5–8%) and High (8.1–11.5%) groups. The three groups were compared with respect to demographic, echocardiographic, intraoperative and postoperative clinical characteristics correlation.

**Results:**

Three patients died during postoperative period. AF occurred in 12 patients (16.6%) after surgery. The incidence of postoperative AF was 15.3% in the lower, 4.4% middle and 57.1% upper group. There was statistically significant correlation between preoperative HbA1C and preoperative stroke, preoperative MI history, Left atrial (LA) size, preoperative levosimendan, preoperative clopidogrel, postoperative AF, postoperative dopamine and dobutamine use, IABP, duration of extubation time, 24-hour chest tube drainage, duration of ICU and hospital mortality. Univariate logistic regression analysis showed significant correlation between postoperative AF and variables like preoperative HbA1c levels, LVEF<30%, history of preoperative MI, preoperative use of levosimendan, preoperative use of clopidogrel, postoperative dopamine, dobutamine adrenaline use, left atrium size, 24-hour chest tube drainage and length of stay in the intensive care unit.

**Conclusion:**

Preoperative HbA1c levels could predict the occurrence of postoperative AF in diabetic patients and may entail to administer protective strategies.

## INTRODUCTION

Atrial fibrillation (AF) is the most common arrhythmic complication of coronary artery bypass grafting surgery (CABG). Depending on the type of cardiac surgery, its incidence varies between 30% and 50%.^1^ The exact factors affecting its incidence are largely unknown. Postoperative AF has been showed to increase both postoperative and 10-year mortality rates, with the latter being increased by 29%.^2^ Some studies have reported that elevated HbA1c levels preceding surgery are linked to the severity of adverse outcomes after CABG.^3^ An elevated HbA1c level is reportedly related to a greater incidence of cardiovascular events.^4^ We aimed to determine the association between preoperative hemoglobin A1c and AF after isolated off-pump coronary bypass grafting (OPCAB).

## METHODS

Seventy-two diabetic patients undergoing OPCAB at our hospital from January 2012 to June 2014 were retrospectively analyzed for AF. The study was approved by the hospital ethic committee. OPCAB was performed in all patients and a shift to cardiopulmonary bypass was not necessary for any patients. Baseline demographic and clinical data were available for all patients, and medical records were accessed for the initial medical data. We divided these patients into three groups (control of diabetic regulation was very good, good and bad) according to the preoperative HbA1c levels. The cutoff points for the groups were selected as follows: Low group; 4.5–5.4% (n =13), Medium group; 5.5–8% (n = 45), and high group: 8.1–11.5% (n =14). Continuous electrocardiographic monitoring via a bedside monitor at the intensive care unit and a telemetry unit at the hospital ward were carried out in all patients after completion of the surgery.

A 12-lead electrocardiogram was taken and evaluated by an experienced physician when the automatic alarm function detected an arrhythmic episode. HbA1c levels were measured before surgery by liquid chromatography. During the bypass surgery, multiple and complete coronary revascularization were attempted at all times, using a composite or sequential grafting in all cases. Arterial grafts in general, and in situ arterial grafts in particular, were used whenever possible. Continuous infusion of diltiazem (0.5-1. 0 µg/kg) was utilized to prevent arterial spasm during and within 24 hours after the operation, followed by oral diltiazem (90 mg/day), acetylsalicylic acid(100 mgr/day) and clopidogrel (75 mg/day) commenced at the next morning.

### Anesthetic & surgical technique

Intravenous fentanyl citrate, midazolam, and vecuronium bromide were employed for anesthesia induction while intravenously administered remifentanyl, vecuronium bromide and low concentrations of inhaled sevoflurane as necessary served for anesthesia maintenance. Heparin was administered to anticoagulated patients after harvesting bypass conduits. The activated clotting time was maintained at more than 250s. The off-pump technique was used for all patients. Intravenous administration of Magnesium (Mg levels were kept over 2 mgr/dl) and potassium were given (K levels were kept over 4 mgr/dl) before pericardiotomy was done for all patients.

### Statistical Analysis

Statistical analysis was performed on SPSS 15.0 for Windows software package with 95% confidence. Independent Sample t-test was used for the parametric features like age, BMI and HbA1c in the men and women. Kruskal Wallis H statistical analysis was used for the comparison between the non-parametric features in continuous variables of more than two groups. Relationships between variables were assessed by Pearson and Spearman correlation analysis. The effects of variables on postoperative AF occurrence were examined using logistic regression analysis. P <0.05 was considered statistically significant.

## RESULTS

**Fig.1:**
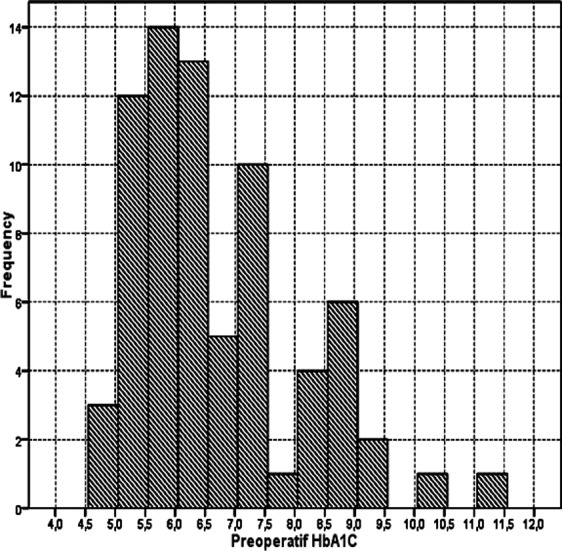
Distribution of Preoperative HbA1c.

Demographic findings of the patient groups can be seen in Table-I. Fig.1 shows the distribution of HbA1c and the median value of HbA1c (25th–75th percentile) was 6.0 (5–7.5). AF occurred in 12 of 72 patients (16.6%) after surgery, most often on postoperative day 2 (75%), with 24.9% of occurrences on postoperative day 3. The incidences of postoperative AF were 15.3% (2/13) in the Low group, 4.4% (2/45) in the Median group, and 57.1% (8/14) in the High group. Three patients died at postoperative period in the High group (hospital mortality, P = 0.000) (Table-I).

**Table-I:** Baseline characteristics & operative variables.

		Preoperative HbA1C			P value

		Low (n=13)	Median (n=45)	High (n=14)
Sex	Male/Female	7/6	26/19	7/7	0,831*
	Age	62,85±7,84	62,4±10,27	66,21±10,21	0,302*
LVEF	(<30)	0 (0)	2 (4,4)	4 (28,6)	0,910*
	(30-50)	10 (76,9)	20 (44,4)	3 (21,4)	
	(>50)	3 (23,1)	23 (51,1)	7 (50)
Chronic renal failure		1 (7,7)	5 (11,1)	1 (7,1)	0,950
Hypertension		10 (76,9)	36 (80)	11 (78,6)	0,922
Previous cerebrovascular disease		3 (23,1)	1 (2,2)	0 (0)	0,011
Chronic pulmonary disease		3 (23,1)	3 (6,7)	2 (14,3)	0,499
Peripheral vascular disease		1 (7,7)	2 (4,4)	0 (0)	0,319
Previous Myocardial infaction		1 (7,7)	12 (26,7)	4 (28,6)	0,213
Left atrial dimension (cm)		3,64±0,4	3,82±0,41	3,99±0,44	0,144
Number of anastomoses		2,92±1,26	2,62±1,3	3,14±1,23	0,245
Number of arterial anastomoses		1,15±0,55	1,27±0,5	1,5±0,52	0,066
Number of vein grafts		2±0,82	1,66±0,55	1,7±0,95	0,462
Preoperative used of Beta- Blockers		1 (7,7)	15 (33,3)	3 (21,4)	0,451
Use of Levosimendan		1 (7,7)	3 (6,7)	5 (35,7)	0,025
Angiotensin converting inhibitors use		4 (30,8)	16 (35,6)	8 (57,1)	0,157
Use of Angiotensin receptor blockers		2 (15,4)	8 (17,8)	1 (7,1)	0,540
Use of Statins		3 (23,1)	14 (31,1)	5 (35,7)	0,482
Use of Oral antidiabetics		11 (84,6)	24 (53,3)	10 (71,4)	0,521
Use of Insulin		1 (7,7)	15 (33,3)	2 (14,3)	0,741
Use of Clopidogrel		2 (15,4)	8 (17,8)	5 (35,7)	0,189
Endartterectomy		2 (15,4)	4 (8,9)	0 (0)	0,150
Use of Intra-aortic balloon pump		0 (0)	0 (0)	4 (28,6)	0,001
Use of insulin during intensive care		7 (53,8)	18 (40)	10 (76,9)	0,243
Postoperative use of Dopamin		4 (30,8)	20 (44,4)	9 (64,3)	0,081*
Postoperative use of Dobutamin		2 (15,4)	2 (4,4)	7 (50)	0,010*
Postoperative use of Adrenalin		1 (7,7)	0 (0)	4 (28,6)	0,028*
Postoperative use of Diltizem		1 (7,7)	8 (17,8)	5 (35,7)	0,066*
Postoperative AF occurrence		0 (0)	4 (8,9)	8 (57,1)	0,000
Post-discharge AF		0 (0)	1 (2,2)	4 (28,6)	0,003
Mortality		0 (0)	0 (0)	3 (21,4)	0,005
Intubation time(hr)		9,08±3,73	7,54±2,56	17,68±29,8	0,072
Chest tube drainage (ml)		457,69±181,25	432,22±183,76	539,29±301,39	0,658
Intensive care unit stay time(hr)		46,15±23,42	39,33±10,97	63,43±31,02	0,006
Mean time to AF occurrence (day)		-	1,5±1	1,38±0,74	0,911

Data are number (percent), mean ± standard deviation, or median [25th–75th percentile]. Low 4.5-5.4%; Median, 5.5-8%; High, >8.%.

In univariate logistic regression analysis between the presence of postoperative AF and variables like LVEF <30 (p=0.015), preoperative MI history (p = 0.004), preoperative use of levosimendan (p = 0.003) and clopidogrel (p = 0.011), postoperative dopamine (P = 0.036), dobutamine (p = 0.000) and adrenaline use (p = 0.004), LA size (P = 0.002), 24-hour chest tube drainage (p = 0.029), length of stay in the ICU (p = 0.003) and preoperative HbA1c (p = 0.000) showed values were statistically significant (p <0.05) (Table-III). Higher HbA1c levels were correlated with intraaortic balloon counter pulsation use (IABP) (p=0.01), preoperative cerebrovascular disease rate (p=0.011), use of preoperative levosimendan usage (p=0.025), use of postoperative dobutamine (p=0.01) and adrenalin (p=0.028) and longer intensive care unit stay (p=0.006). Correlation analysis showed that there is statistically significant correlation between preoperative HbA1C and occurrences of SVO (p = 0.020), postoperative AF with preoperative MI history (p = 0.002), Left atrial (LA) size (p = 0.000), preoperative use of levosimendan (p = 0.001), preoperative use of clopidogrel (p = 0.006), postoperative dopamine (p = 0.000) and dobutamine use (p = 0.000), IABP usage (p = 0.011), duration of extubation (p = 0.011), 24 hour chest tube drainage (p = 0.011), duration of ICU (p = 0.01) and hospital mortality (p = 0.000) (p <0.05) (Table-II).

**Table-II:** Correlation analysis of preoperative & postoperative variables with HbA1c values associated with AF.

	Preoperative HbA1C		Postoperative AF	

	r	p	r	p
Previous cerebrovascular disease	-0,273	0,020	-0,108	0,364
Previous Myocardial infaction	0,057	0,632	0,366	0,002
Left atrial dimension (cm)	0,151	0,204	0,441	0,000
Levosimendan	0,201	0,090	0,394	0,001
Clopidogrel	0,089	0,458	0,321	0,006
Postoperative used of Dopamin	0,081	0,501	0,262	0,026
Postoperative used of Dobutamin	0,232	0,049	0,535	0,000
Postoperative used of Adrenalin	0,253	0,032	0,464	0,000
Use of Intra-aortic balloon pump	0,361	0,002	0,542	0,000
Intubation time(hr)	0,167	0,161	0,300	0,011
Chest tube drainage (ml)	0,113	0,346	0,276	0,019
Intensive care unit stay time(hr)	0,349	0,003	0,472	0,000
Mortality	0,303	0,010	0,466	0,000

**Table-III:** All variables with the presence of postoperative AF in univariate logistic regression analysis.

		AF+ (n=12)	AF – (n=60)	Odds Ratio (95% C.I.)	p
Sex	M/F	4/8	28/32	1,75 (0,48-6,44)	0,400
	Age	65,92±11,1	62,68±9,6	1,04 (0,97-1,11)	0,299
LVEF	(>50)	5 (41,7)	28 (46,7)	Referance	0,015
	(30-50)	3 (25)	30 (50)	0,56 (0,12-2,56)	0,455
	(<30)	4 (33,3)	2 (3,3)	11,2 (1,6-78,4)	0,015
Chronic renal failure		1 (8,3)	6 (10)	0,82 (0,09-7,49)	0,859
Hypertension		11 (91,7)	46 (76,7)	3,35 (0,4-28,25)	0,267
Previous cerebrovascular disease		- (-)	4 (6,7)	0 (0-)	0,999
Chronic pulmonary disease		3 (25)	5 (8,3)	3,67 (0,74-18,08)	0,110
Peripheral vascular disease		- (-)	3 (5)	0 (0-)	0,999
Previous Myocardial infaction		7 (58,3)	10 (16,7)	7 (1,84-26,56)	0,004
Preoperative used of Beta- Blockers		5 (41,7)	14 (23,3)	2,35 (0,64-8,56)	0,196
Levosimendan		5 (41,7)	4 (6,7)	10 (2,16-46,26)	0,003
Angiotensin converting inhibitors		6 (50)	22 (36,7)	1,73 (0,5-6,01)	0,391
Angiotensin receptor blockers		1 (8,3)	10 (16,7)	0,45 (0,05-3,93)	0,474
Statins		4 (33,3)	18 (30)	1,17 (0,31-4,37)	0,819
Oral antidiabetics		7 (58,3)	38 (63,3)	0,81 (0,23-2,86)	0,744
Insulin		3 (25)	15 (25)	1 (0,24-4,18)	1,000
Clopidogrel		6 (50)	9 (15)	5,67 (1,49-21,54)	0,011
Endartterectomy		- (-)	6 (10)	0 (0-.)	0,999
Postoperative use of Dopamin		9 (75)	24 (40)	4,5 (1,1-18,34)	0,036
Postoperative use of Dobutamin		7 (58,3)	4 (6,7)	19,6 (4,24-90,67)	0,000
Postoperative use of Adrenalin		4 (33,3)	1 (1,7)	29,5 (2,92-297,9)	0,004
Postoperative use of Diltizem		1 (8,3)	13 (21,7)	0,33 (0,04-2,79)	0,308
Use of Intra-aortic balloon pump		4 (33,3)	- (-)		0,999
Use of insulin during intensive care		9 (75)	26 (44,1)	3,81 (0,94-15,5)	0,062
Post-discharge AF		5 (41,7)	- (-)		0,999
Hospital Mortality		3 (25)	- (-)		0,999
Left atrial dimension (cm)		4,23±0,38	3,73±0,39	21,84 (3,25-146,71)	0,002
Number of anastomoses		2,92±1,24	2,75±1,3	1,1 (0,69-1,77)	0,679
Number of arterial anastomoses		1,25±0,45	1,3±0,53	0,82 (0,23-2,94)	0,758
Number of vein grafts		2±0,93	1,68±0,65	1,9 (0,64-5,64)	0,245
Intubation time(hr)		18,83±32,25	7,98±2,86	1,13 (0,96-1,34)	0,153
Chest tube drainage (ml)		587,5±296,28	431,67±182,95	1 (1-1,01)	0,029
Intensive care unit stay time(hr)		67±32,14	40,9±14,51	1,05 (1,02-1,09)	0,003
Preoperative HbA1C		8,58±1,38	6,31±1,06	3,92 (1,92-7,99)	0,000

Values are odds ratio (95% confidence interval), LV, left ventricle; EF, Ejection fraction.

Corrected Bonferroni Mann Whitney U test was used to find out the correlation of HbA1c level and several clinical conditions like the use of dopamine and ICU durations between medial and high groups (p = 0.008, p=0.001 p<0.016 respectively) (Table-I). The mortality was significantly higher in High group (n=3, 100%) compared to low and median groups.

When examined all variables of preoperative HbA1c groups, statistically significant differences were found between groups of postoperative AF, AF after discharge and hospital mortality (p<0, 05) (Table-I).

## DISCUSSION

Association of OPCAB surgery with decreased incidence of AF was shown in a meta analysis and provided evidence that OPCAB surgery may be associated with a reduced incidence of postoperative AF than on-pump CPB techniques in a generalized population. The off-pump and on-pump groups had postoperative AF incidences of 19% (1612/8265) and 24% (1976/8240).^5^ Our study revealed that AF complicated 16.6% of off-pump operations. Limited number of patients in our study can be shown to be the main reason of this low rates. In a prospective cohort study of 3089 patients with and without diabetes, Halkos et al.^6^ showed a significant correlation between HbA1c and in-hospital mortality and postoperative morbidity. The rates of postoperative complications, e.g. renal failure and cerebrovascular accident, were higher in patients with HbA1c ≥ 8.6%. AF occurred in 20.9% of patients having an HbA1c of less than 7.0% vs. 15.1% in those with an HbA1c level equal to or greater than 7.0%. Patients with a poor glycemic control evidenced by an HbA1c ≥7% had more complications than those with an HbA1c of less than 7%. Similarly, in 101 diabetic subjects operated with off-pump CABG, Matsuura et al.^7^ found a postoperative AF rate of 29.7% for an HbA1c of less than 6.5% and 22.2% for an HbA1c that was greater than 6.5%. In our study 72 diabetic patients undergoing OPCAB were retrospectively analyzed. We divided these patients into three groups according to the preoperative HbA1c levels (Low group; 4.5–5.4%, Medium group; 5.5–8%, and high group: 8.1–11.5%). Corrected Bonferroni Mann Whitney U test was used to find out the correlation of HbA1c level and several clinical conditions like the use of dopamine, ICU durations and mortality rates between medial and high groups. As a reason for this result, all of the patients in a low group LVEF values greater than 30%. However, Matsuura et al the two groups did not differ significantly with regard to early and late postoperative mortality, wound dehiscence, number of anastomosis, use of bilateral internal thoracic arteries, and patency rates. On the other hand, in 805 diabetic subjects operated with OPCAB surgery, Kinoshita et al.^8^ reported that the lower group, the middle group, and the upper group had postoperative AF rates of 28.3%, 17.4%, and 12.5%, respectively. Postoperative AF was less likely when an elevated preoperative HbA1c was present. Our study demonstrated that the lower group, the middle group, and the upper group had postoperative AF incidences of 15.3%, 4.4%, and 57.1%, respectively. The risk of postoperative AF was greater with an elevated preoperative HbA1c. Dublin et al.^9^ demonstrated an elevated risk in parallel with HbA1c level in a population-based case control study conducted in 1410 patients with newly-diagnosed AF. Another population-based study in 75-year-old subjects similarly showed higher HbA1c levels in patients who developed AF.^10^ Diabetics with higher HbA1c levels also suffer a higher rate of perioperative myocardial infarction.^6^,^11^. Our study also showed similar results in that higher HbA1c levels significantly associated with risk of death. Faritous et al.^12^ demonstrated that a higher incidence of IABP, massive bleeding, and multi-organ failure result from higher HbA1c levels. Morbidity, but not mortality, is increased by elevated HbA1c levels following CABG. We showed that elevated HbA1c level increased the likelihood of IABP use (p=0.01), rate of preoperative cerebrovascular disease (P=0.011), use of preoperative levosimendan (P=0.025), use of postoperative dobutamine (p=0.01) in conjunction with adrenaline (p=0.028), longer intensive care unit stay (p=0.006), postoperative and post-discharge AF, and mortality rate (p<0, 05). Goksedef et al.^13^ reported that in 150 diabetic and non-diabetic patients enrolled in a prospective fashion the rates of postoperative mediastinitis and local sternal infection (P = 0.8) were not significantly altered by an HbA1c level of >7%. On the other hand, Sato et al.^14^ in a prospective study of 273 diabetic and non-diabetic patients, demonstrated that diabetics with a HbA1c greater than 6.5% suffered significantly more postoperative complications (minor infections and a more labor-intensive hospital stay). Alserius et al.^15^ Prospectively investigated the relationship between HbA1c level and infection and mortality rates in 605 subjects. HbA1c level greater than 6% was associated with significantly increased incidence of superficial sternal wound infections and mortality.

### Limitations of the study

First, off-pump technique was performed at a single center. Second, it was not a randomized controlled study. Third, it solely used preoperative laboratory data including HbA1c level but not assessed postoperative HbA1c alterations.

## CONCLUSION

Our study showed that preoperative HbA1c level was associated with AF in diabetic patients undergoing OPCAB. In conclusion, preoperative HbA1c could be predicts the occurrence of postoperative AF after isolated off-pump CABG.
